# Sex, BMI and age differences in metabolic syndrome: the Dutch Lifelines Cohort Study

**DOI:** 10.1530/EC-17-0011

**Published:** 2017-04-18

**Authors:** Sandra N Slagter, Robert P van Waateringe, André P van Beek, Melanie M van der Klauw, Bruce H R Wolffenbuttel, Jana V van Vliet-Ostaptchouk

**Affiliations:** Department of EndocrinologyUniversity of Groningen, University Medical Center Groningen, Groningen, The Netherlands

**Keywords:** metabolic syndrome, blood pressure, age adjusted, population based, sex, BMI

## Abstract

**Introduction:**

To evaluate the prevalence of metabolic syndrome (MetS) and its individual components within sex-, body mass index (BMI)- and age combined clusters. In addition, we used the age-adjusted blood pressure thresholds to demonstrate the effect on the prevalence of MetS and elevated blood pressure.

**Subjects and methods:**

Cross-sectional data from 74,531 Western European participants, aged 18–79 years, were used from the Dutch Lifelines Cohort Study. MetS was defined according to the revised NCEP-ATPIII. Age-adjusted blood pressure thresholds were defined as recommended by the eight reports of the Joint National Committee (≥140/90 mmHg for those aged <60 years, and ≥150/90 mmHg for those aged ≥60 years).

**Results:**

19.2% men and 12.1% women had MetS. MetS prevalence increased with BMI and age. Independent of BMI, abdominal obesity dominated MetS prevalence especially in women, while elevated blood pressure was already highly prevalent among young men. Applying age-adjusted blood pressure thresholds resulted in a 0.2–11.9% prevalence drop in MetS and 6.0–36.3% prevalence drop in elevated blood pressure, within the combined sex, BMI and age clusters.

**Conclusions:**

We observed a gender disparity with age and BMI for the prevalence of MetS and, especially, abdominal obesity and elevated blood pressure. The strict threshold level for elevated blood pressure in the revised NCEP-ATPIII, results in an overestimation of MetS prevalence.

## Introduction

The metabolic syndrome (MetS) is nowadays frequently used to identify individuals at higher risk for future type 2 diabetes (T2D) and cardiovascular disease (CVD) (1). Recognized metabolic risk components are abdominal obesity, dyslipidaemia, elevated blood pressure and elevated fasting glucose. However, the estimated prevalence of MetS differs between various populations, because variations exist in the frequencies of metabolic risk components (2). It has also been reported that the prevalence of each metabolic risk component differs with sex (3, 4, 5). Especially abdominal obesity is more common in women (2, 4, 5). Whether the sex differences in the MetS features persist within different body mass index (BMI) classes and across different age groups, is unclear.

Previously, we observed that besides abdominal obesity, elevated blood pressure was the most common abnormality contributing to the prevalence of MetS, within all BMI classes (6, 7). Elevated blood pressure is also very common among the elderly, and many studies have described a gradual increase of blood pressure with increasing age (8, 9, 10). The rise in systolic blood pressure continues throughout life in contrast to diastolic blood pressure, which shows a reversed U-shaped trend with age (11). It can, therefore, be argued that the defining value for elevated blood pressure used in the revised NCEP-ATPIII definition for MetS (systolic blood pressure ≥130 or diastolic blood pressure ≥85 mmHg) is too low for an elderly population, and may lead to overestimation of the MetS prevalence. An earlier paper has suggested that the blood pressure level used in the definition of MetS should be adjusted to age (8). In addition, recent guidelines on the treatment of elevated blood pressure indicate higher and age-adjusted blood pressure levels to start either a lifestyle or medical intervention (12, 13). Harmonisation of diagnostic criteria would greatly benefit the implementation of MetS in clinical practice.

Despite the prevalence of MetS is well known in various populations, there is no in-depth information available about the prevalence of MetS and the individual components within particular combined subgroups of sex, BMI and age. The Lifelines cohort is the largest population-based study in the Netherlands and therefore particularly suitable to evaluate these detailed prevalence estimates. Our second aim was to demonstrate the influence of age-adjusted blood pressure thresholds on the prevalence estimates of MetS and elevated blood pressure.

## Methods

### The Lifelines Cohort Study

Lifelines is a population-based cohort study examining in a unique three-generation design the health and health-related behaviours of persons living in the North of the Netherlands. The adult population participating in Lifelines was found to be broadly representative for the adults living in the three northern provinces of the Netherlands (14). Between 2006 and 2013, different recruitment strategies were adopted (recruitment of an index population (aged 25–49 years) via general practitioners, subsequent inclusion of their family members, and online self-registration) which resulted in a low risk of selection bias (15). The Lifelines Cohort Study is conducted according to the principles of the Declaration of Helsinki and in accordance with the research code of the University Medical Center Groningen (UMCG). Before study entrance, all participants signed an informed consent. The study was approved by the medical ethics review committee of the UMCG.

For this study, we used cross-sectional data, collected between 2006 and 2013, of subjects from Western European descendent (according to self-reported information in the questionnaire) and aged ≥18 and <80 years (*N* = 92,409). We excluded individuals who had no verified data on medication use or missing data on variables needed to calculate the body mass index or on the variables used to diagnose MetS. A total of 74,531 individuals were included in the study.

### Clinical measurements

A standardized protocol was used to obtain blood pressure and anthropometric measurements such as height, weight and waist circumference. Blood pressure was measured every minute during a period of 10 min with an automated DINAMAP Monitor (GE Healthcare). The average of the final three readings was recorded for systolic and diastolic blood pressure. Anthropometric measurements were taken in light clothing and without shoes. Body weight was measured to the nearest 0.1 kg. Height and waist circumference were measured to the nearest 0.5 cm. Waist circumference was measured in standing position with a tape measure all around the body, at the level midway between the lower rib margin and the iliac crest. Body weight and height were used to calculate BMI (weight (kg)/height (m^2^)), which was categorized as normal weight (<25 kg/m^2^), overweight (25–30 kg/m^2^) and obesity (≥30 kg/m^2^).

Blood was collected in the fasting state, between 08:00 and 10:00 in the morning. On the same day, serum levels of HDL cholesterol were measured, using an enzymatic colorimetric method, and triglycerides, using a colorimetric UV method on a Roche Modular P chemistry analyzer (Roche). Fasting blood glucose was measured using a hexokinase method.

### Definitions of metabolic syndrome and metabolic risk components

According to the revised NCEP-ATPIII (R-ATPIII) (16), at least three out of the five metabolic risk components need to be present to diagnose MetS. These metabolic risk components include (1) systolic blood pressure ≥130 mmHg and/or diastolic blood pressure ≥85 mmHg and/or use of antihypertensive drugs; (2) fasting blood glucose ≥5.6 mmol/L and/or use of blood glucose-lowering medication and/or diagnosis of T2D; (3) HDL cholesterol levels <1.03 mmol/L in men and <1.30 mmol/L in women and/or use of lipid-lowering medication influencing these parameters; (4) triglyceride levels ≥1.70 mmol/L and/or use of triglyceride-lowering medication and (5) waist circumference ≥102 cm in men and ≥88 cm in women.

According to the most recent hypertension guideline from the eighth report of the Joint National Committee (JNC 8, 2014), non-diabetic individuals between 18 and 60 years should be treated to a target blood pressure <140/90 mmHg and individuals ≥60 years to a target blood pressure of <150/90 mmHg. Accordingly, age-adjusted thresholds for elevated blood pressure were considered at: (1) systolic blood pressure ≥140 and/or diastolic blood pressure ≥90 mmHg for those aged <60 years, and (2) systolic blood pressure ≥150 and/or diastolic blood pressure ≥90 mmHg for those aged ≥60 years (12). MetS defined by the age-adjusted thresholds for blood pressure are referred to as ‘revised NCEP-ATPIII updated’ (R-ATPIII updated).

All medications used by participants were self-reported and classified according to the Anatomical Therapeutic Chemical (ATC) classification system. Diagnosis of T2D was based on self-report and verified with self-reported medication use. Newly diagnosed T2D was based on a single fasting blood glucose level ≥7.0 mmol/L. A CVD history was defined as self-reported previously sustained myocardial infarction, stroke or vascular intervention.

### Demographic and lifestyle factors

Based on the participants’ responses to the self-administered questionnaires, data were assessed on education level, smoking, alcohol consumption and physical activity. Education level was categorized as low (no formal education, only primary school or intermediate vocational education), medium (higher secondary education) or high (higher vocational education and university). Smoking status was defined as non-smoker, former smoker and current smoker (including the use of cigarettes, cigarillos, cigars and pipe tobacco) (17). Alcohol consumption was defined as non-drinker, ≤2 drinks/day and >2 drinks/day (17). A physically active lifestyle was based on the question ‘Being active for at least half an hour a day’.

### Data analysis

The prevalence of MetS (according to the R-ATPIII and R-ATPIII updated) and each metabolic risk factor were reported in subgroups that were defined by sex, BMI (normal weight, overweight and obese) and age decades (18–29, 30–39, 40–49, 50–59, 60–69 and 70–79 years). Results are expressed as counts and/or proportions (%). All data analyses were conducted using IBM SPSS Statistics, version 23 (IBM Corporation). In our analysis, we chose to focus on absolute differences and not on statistical significance, because the large study sample of Lifelines may produce low *P* values even when absolute differences are small.

## Results

In the present study, data of 74,531 individuals were used, including 32,731 (43.9%) men (mean age 45 ± 13 years) and 41,800 (56.1%) women (mean age 45 ± 12 years). Among the male participants, 12,691 (38.8%) were normal weight, 15,677 (47.9%) were overweight and 4363 (13.3%) were obese. Among female participants, these numbers were 21,460 (51.3%), 13,893 (33.2%) and 6447 (15.5%), respectively. Clinical, demographic and lifestyle characteristics of the study population can be found in Table 1. Alcohol use (especially >2 drinks/day) and smoking were more common in men than in women. Among men and women, only 23.7% and 25.5% reported to have an active lifestyle, respectively. In Supplementary Table 1A, B and C (see section on supplementary data given at the end of this article), clinical characteristics are depicted for the sex, age and BMI stratified samples. The prevalence of T2D and CVD history increased with age and BMI. Among older adults (≥60 years), the prevalence of T2D and CVD history were respectively, 9.4% and 9.1% in men and 7.5% and 3.1% in women.

**Table 1:** Clinical characteristics of the study population.

	**Men** (*N* = 32,731)	**Women** (*N* = 41,800)
Age (years)	45.2 ± 12.7	44.9 ± 12.5
Weight (kg)	87.6 ± 13.1	73.6 ± 13.5
BMI (m/kg^2^)	26.3 ± 3.6	25.7 ± 4.6
Waist circumference (cm)	94.9 ± 10.6	86.6 ± 12.0
Systolic BP (mmHg)	131 ± 14	122 ± 15
Diastolic BP (mmHg)	76 ± 9	72 ± 9
HDL-cholesterol (mmol/L)	1.30 ± 0.32	1.61 ± 0.39
Triglycerides (mmol/L)	1.39 (0.83–1.65)	1.01 (0.65–1.20)
Fasting blood glucose (mmol/L)	5.2 ± 0.8	4.9 ± 0.7
Use of antihypertensive drugs (%)	9.0	9.1
Type 2 diabetes (%)	2.7	1.8
CVD history (%)	2.1	0.8
Education level (%)		
Low	30.3	31.3
Middle	38.2	39.9
High	31.5	28.8
Smoking (%)		
Non-smoker	43.7	48.0
Former smoker	32.6	32.1
Current smoker	23.7	19.9
Alcohol (%)		
Non	8.5	22.5
≤2 drinks/day	76.0	73.8
>2 drinks/day	15.6	3.7
Physical active lifestyle (30 min/day)	23.7	25.5

Available data in men and women, respectively: education level – 32,108 and 40,653; smoking – 32,189 and 41,075; alcohol – 31,347 and 40,066; physical activity – 30,126 and 38,050.

BMI, body mass index; BP, blood pressure; CVD, cardiovascular disease; HDL-cholesterol, high density lipoprotein cholesterol.

### The prevalence of MetS, according to the two operating definitions

The age-, sex- and BMI-specific prevalence of MetS according to the R-ATPIII and R-ATPIII updated criteria are shown in Table 2. In both men and women, the prevalence of MetS increased with age in all BMI classes, irrespective of the used cut-offs for blood pressure. Also, the number of MetS components increased with age (Table 3). In general, MetS was more common in men than in women. Only in normal weight women ≥60 years and overweight women ≥70 years, MetS prevalence exceeded that of men (Table 2). When the age-adjusted blood pressure thresholds were used to define MetS (R-ATPIII updated), the percentage of subjects with MetS decreased with 0.9–11.9% in men and 0.2–8.6% in women (Table 2).

**Table 2:** Percentage of subjects with metabolic syndrome, according to clustered subgroups of sex, BMI and age.

	**Men**			**Women**		
**Age** (years)	No. of subjects	R-ATPIII	R-ATPIII updated	No. of subjects	R-ATPIII	R-ATPIII updated
Normal weight						
18–29	2507	1.6	0.7	3474	0.5	0.3
30–39	3244	2.9	1.5	5260	1.1	0.7
40–49	4002	4.2	2.6	7833	1.8	1.4
50–59	1600	4.8	2.5	3006	3.6	2.3
60–69	976	5.2	3.8	1453	6.3	4.8
70–79	362	7.7	6.1	434	11.8	10.4
Overweight						
18–29	1154	8.9	5.2	1142	6.2	3.2
30–39	3262	16.2	10.6	2684	6.9	4.6
40–49	5974	20.8	15.7	5014	13.1	10.1
50–59	2636	22.3	15.6	2519	17.0	12.1
60–69	1893	27.0	21.0	1808	26.2	21.6
70–79	758	31.5	28.5	726	35.5	30.3
Obese						
18–29	241	47.7	38.6	493	22.7	14.6
30–39	857	54.1	42.2	1323	25.5	19.0
40–49	1734	59.9	51.5	2427	37.5	31.0
50–59	780	60.8	50.6	1009	48.0	39.4
60–69	578	68.5	57.8	823	55.5	46.9
70–79	173	68.8	63.6	372	59.4	54.0

R-ATPIII updated is based on the age-adjusted blood pressure cut-offs, i.e. ≥140 mmHg (systolic) and/or ≥90 mmHg (diastolic) for those aged <60 years, and ≥150 mmHg (systolic) and/or ≥90 mmHg (diastolic) for those aged ≥60 years.

**Table 3:** Prevalence of having one to five MetS components by sex and age groups according to the R-ATPIII.

	18–29	30–39	40–49	50–59	60–69	70–79
Men						
Number of subjects	3902	7363	11,710	5016	3447	1293
None	47.3	33.9	26.7	22.9	14.7	7.8
One	34.0	33.3	31.3	30.9	32.2	34.3
Two	12.1	18.1	21.1	23.6	25.3	28.0
Three	4.9	9.9	12.6	13.7	18.2	18.4
Four	1.4	4.0	6.5	6.8	6.8	8.2
All five	0.3	0.9	1.9	2.2	2.8	3.2
Women						
Number of subjects	5109	9267	15,274	6534	4084	1532
None	54.1	47.9	40.5	30.2	14.9	6.9
One	29.0	30.7	30.5	33.3	30.3	22.7
Two	12.9	15.1	17.9	20.9	29.7	35.8
Three	3.3	4.9	7.6	10.0	14.9	20.4
Four	0.5	1.1	2.9	4.2	7.7	10.3
All five	0.1	0.2	0.7	1.5	2.5	3.9

### The prevalence of the individual metabolic risk factors in the total population

**Figure 1:**
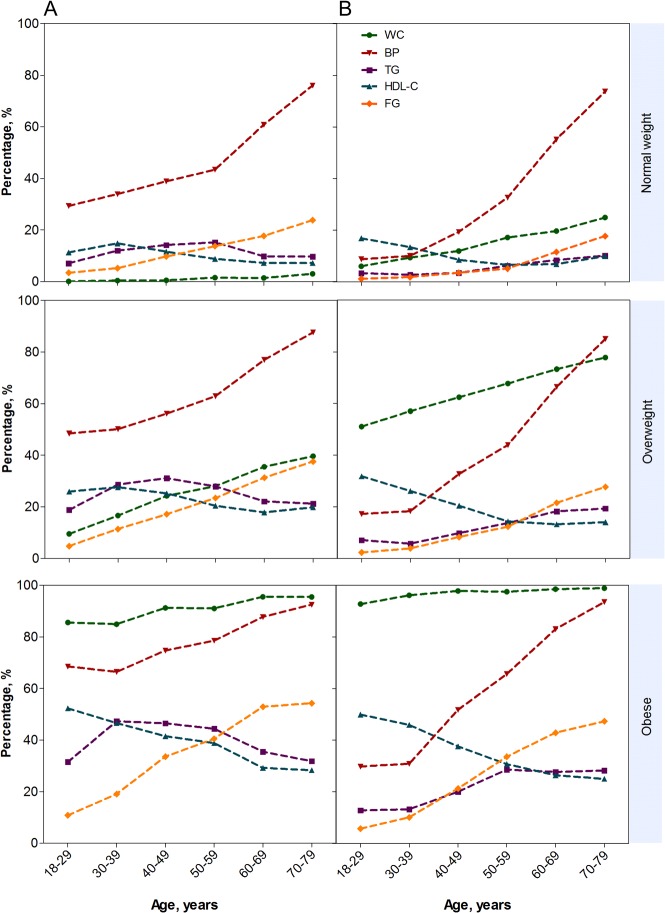
Prevalence of the metabolic syndrome components in the total population. Left panel A: men, and right panel B: women. BP, blood pressure; FG, fasting glucose; HDL-C, high density lipoprotein cholesterol; TG, triglycerides; WC, waist circumference.

Figure 1 illustrates the prevalence of the individual MetS components, applying the cut-offs for the individual metabolic risk factors as recommended by the R-ATPIII. Exact numbers of the prevalence estimates can be found in Supplementary Table 2A, B and C. In men below the age of 60 years, the most common MetS component was elevated blood pressure (49.6%), followed by increased triglycerides (24.1%) and decreased HDL cholesterol (22.1%). In women below the age of 60 years, abdominal obesity (39.0%), elevated blood pressure (25.2%) and decreased HDL cholesterol (18.5%) were the most common MetS components. However, in older adults (≥60 years), the sex differences in the various MetS components were diminished. In both sexes, elevated blood pressure (75.9% in men and 69.2% in women), abdominal obesity (35.6% in men and 60.9% in women) and impaired fasting glucose (32.2% in men and 23.8% in women) were the most prevalent.

#### Elevated blood pressure

**Figure 2:**
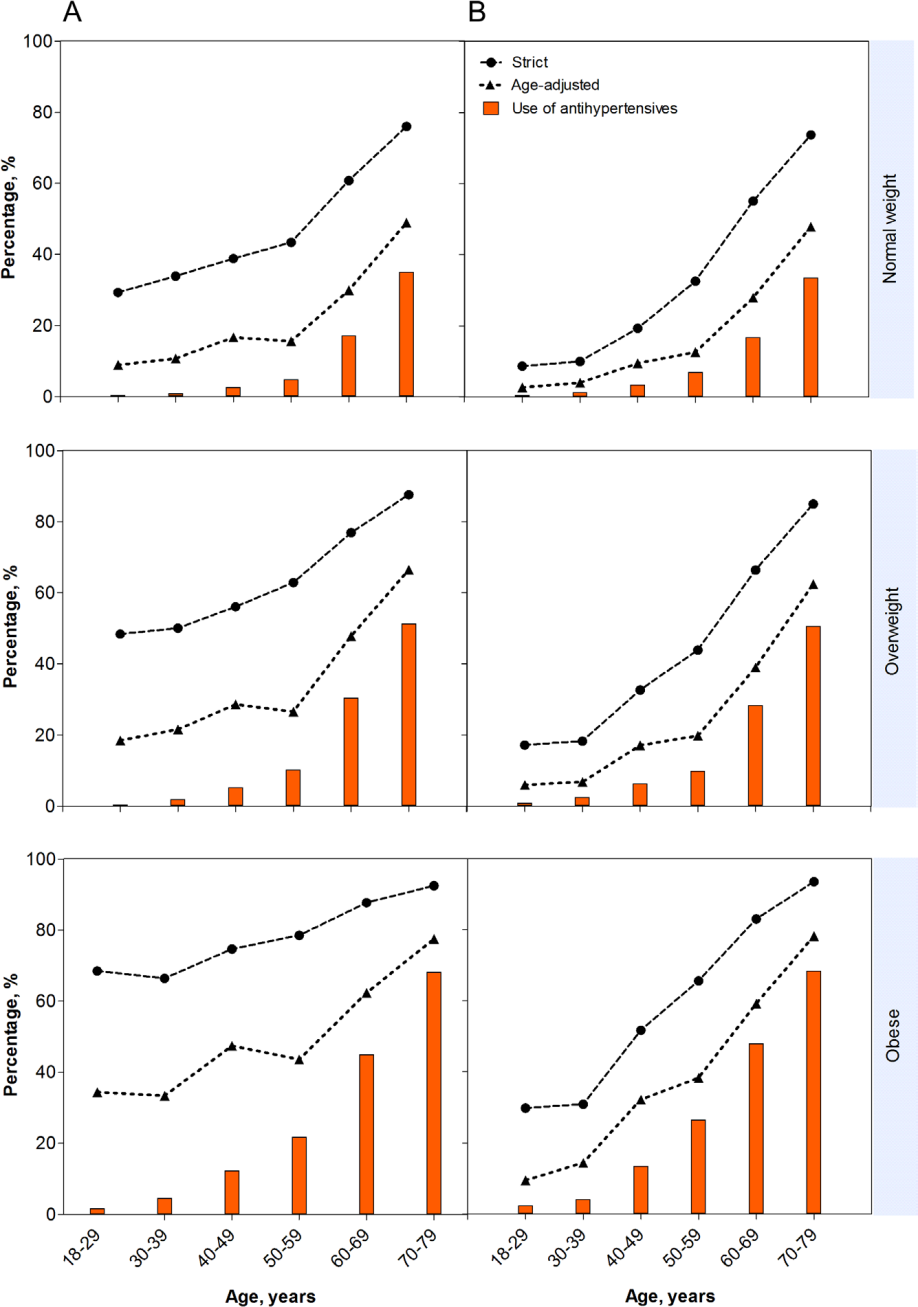
Prevalence of elevated blood pressure, according to the strict and age-adjusted thresholds, including antihypertensive drug use. Left panel A: men, and right panel B: women. BP, blood pressure. Strict blood pressure values are ≥130 mmHg (systolic) or ≥85 mmHg (diastolic) (including those using antihypertensive drugs). Age-adjusted blood pressure values are ≥140 mmHg (systolic) or ≥90 mmHg (diastolic) for those aged <60 years, and ≥150 mmHg (systolic) or ≥90 mmHg (diastolic) for those aged ≥60 years (including those using antihypertensive drugs).

The MetS component ‘elevated blood pressure’ showed the most pronounced increase with age. Across the entire cohort, the prevalence of elevated blood pressure (≥130/85 mmHg, including participants receiving antihypertensive drugs) increased from 23.3% in the youngest age group (18–29 years) to 84.4% in the oldest age group (70–79 years). In men below the age of 60 years, elevated blood pressure was present in a much higher percentage compared to women (independent of BMI). Among individuals aged ≥60 years, the percentages of men and women with elevated blood pressure were roughly similar (Figs 1 and 2).

In Fig. 2, the prevalence of elevated blood pressure is displayed for the age-adjusted blood pressure thresholds, together with the strict threshold of the R-ATPIII for comparison. The prevalence estimates also include those using antihypertensive drugs. The use of age-adjusted blood pressure thresholds resulted in a large reduction of subjects fulfilling the criteria ‘elevated blood pressure’ compared to when the strict threshold for elevated blood pressure was used. This was most pronounced among younger men (<60 years), where the prevalence of elevated blood pressure dropped with 20.4–36.3%, depending on the age and BMI group. Supplementary Table 1A, B and C depict the absolute blood pressure levels in the various age and BMI groups, and the percentage of participants using antihypertensive drugs. With increasing age and higher BMI, the use of antihypertensive drugs increased, and this was comparable between men and women. The age-adjusted prevalence estimates of elevated blood pressure were closer to the estimates for antihypertensive drug use. In other words, the ratio of those having an elevated blood pressure based on their measured blood pressure values vs the use of antihypertensive drugs decreased.

#### Abdominal obesity

Prevalence of abdominal obesity became higher with increasing age and was higher among women than men. In normal weight women, the prevalence of abdominal obesity increased from 6.0% to 24.9% with age. This percentage was much lower among normal weight men, namely 0.1–3.0%. The sex difference was also present in overweight (9.5–39.6% in men and 51.0–77.8% in women) and obese individuals, although among obese individuals essentially all had a waist circumference above the defined cut-offs for abdominal obesity (Fig. 1).

#### Dyslipidaemia and impaired fasting glucose

The prevalence of decreased HDL cholesterol gradually fell with increasing age in both men and women (Fig. 1). In contrast, the prevalence of elevated triglycerides became higher with increasing age among women, while in men, there was a reversed U-shaped trend. In both men and women, the prevalence of impaired fasting glucose increased with age as well, being most pronounced in overweight and obese individuals. Only from the age of 60 years onwards, impaired fasting glucose became one of the three most prevalent MetS components (Fig. 1).

## Discussion

In Western European individuals living in the Netherlands, the prevalence of MetS risk factors differed by sex, age and BMI. Elevated blood pressure and abdominal obesity were the two most frequently present risk factors, and their contribution to the diagnosis of MetS greatly overrides the other three components. Furthermore, the age-adjusted thresholds for elevated blood pressure better approximated the treatment of hypertension in clinical practice.

### Prevalence of MetS (components)

This is one of the largest studies in the Netherlands, in which the prevalence of MetS was meticulously assessed. Similar to other observations, we found that the prevalence of MetS increases with age, up to the seventh age decade (1, 18, 19), and that it is higher in men than in women (2, 4, 5). Data from NHANES III (1988–1994) showed, however, that prevalence of MetS in women exceeded that of men, when individuals older than 50 years of age were evaluated (4). In our population, we observed a higher prevalence of MetS only in normal weight women aged ≥60 years and overweight women aged ≥70 years compared with men from the same age and BMI group. The difference in prevalence of MetS between men and women may be related to differences in body fat distribution: men have more visceral and hepatic fat, whereas women have more total body fat (20). Differences in distribution of fat with age (total fat and visceral fat) and the cardiometabolic effects of menopause may explain the diminished sex difference in MetS prevalence seen with older age (20, 21). Furthermore, men do smoke more tobacco and drink more alcohol then women. These two substances are both associated with the development of MetS (17).

In some studies, prevalence estimates for MetS are found to plateau or drop off after the sixth or seventh age decade in both sexes (22, 23), or only in men (24, 25, 26, 27). This observation might be due to a survival effect or participation bias, as individuals prone to obesity-related morbidity and mortality have already died or decline to participate in a study (28). While it may also depend on the definition used for MetS (1, 22), even if the same definition was used, different trends were observed between countries (7, 19, 26). This underpins the importance of estimating the country-specific prevalence of MetS.

The observed trend of increasing MetS prevalence with age can be explained by the large number of people developing metabolic complications by the time they are aged ≥60 years (i.e. more than 85% of the individuals have at least one metabolic risk factor). Due to the age-related rises of blood pressure, abdominal obesity and glucose a more similar make-up of MetS was seen in the elderly, whereas in younger people, the MetS profile was more heterogeneous and differed more by sex. As reported previously, abdominal obesity was already highly prevalent in younger women and much more common than in men (2, 4, 5), which is interesting since in general, women store more fat on the hips and thighs (29). Moreover, a cross-sectional study among German Caucasians showed that the ≥88 cm cut-off for abdominal obesity was too low for capturing CVD risk in women, while a lower cut-off of ≥94 cm seemed appropriate in men (30). In young men, we found that a large proportion had an elevated blood pressure, namely 42.3% below the age of 40 years (≥130/85 mmHg). This is much higher than the 24.1% found in 20- to 39-year-old men from the NHANES 2003–2006 study (3). This finding may suggest that, across the entire lifespan, blood pressure has a greater relative importance in the development of MetS in men than in women. One possible explanation for this finding may be the high alcohol consumption in men. In our study, below the age of 40 years, 15.9% of men consume >2 drinks/day (mean 20.7 g ethanol), whereas this is only 2.2% in women (mean 16.4 g ethanol). In our previous study, alcohol consumption showed a ‘J-shaped’ relationship with blood pressure (17). There are consistent data showing that alcohol consumption increases blood pressure and risk for hypertension. However, in men already light-to-moderate alcohol intake seems to increase risk for hypertension, whereas in women, at these levels, a potential reduced risk of hypertension was found (31, 32). Further research is needed to clarify why already a large group of young men have a blood pressure above ‘high normal’.

The clinical utility of MetS has been criticized for quit some years (33, 34). Criticism is related to the predictive value of MetS for CVD. MetS is found to have no greater predictive value for CVD compared to the individual components (35). Furthermore, all MetS components are weighted equally while it is clear that some risk factors are more important for risk prediction. Also, continuous variables are dichotomized and MetS is operationalized as a combination of three or more of the five components, which results in a loss of predictive power (36). In the current R-ATPIII definition, only blood pressure and fasting glucose are used for targeted risk factor interventions in clinical practice. Though, interventions are seldom started at the levels proposed by the R-ATPIII. Below we will discuss the suggested threshold for the high prevalent ‘elevated blood pressure’ feature in the MetS definition.

### ‘Elevated’ blood pressure in different age groups

Compared to the other MetS components, the prevalence of elevated blood pressure was remarkably high in our study. A threshold of ≥130/85 mmHg seems very strict, especially for the older subjects where the natural course of blood pressure changes with ageing is not taken into account (37). Since approximately half of the deaths from stroke or CVD is attributable to hypertension (38), early screening and diagnosis of hypertension is important. However, the optimal threshold of blood pressure for intervention remains disputable, especially in the elderly (39, 40).

Over the last decades, several guidelines have tried to define the optimal cut-off levels for treatment of hypertension with lifestyle adjustment and medication. In the most recent JNC 8 treatment guideline for hypertension, it is advised to aim for a blood pressure <140/90 mmHg in non-diabetic adults (<60 years), whereas blood pressure values <150/90 mmHg were advised for elderly (≥60 years) (12). In the current study, we applied both the very strict blood pressure threshold from the R-ATPIII as well as these age-adjusted thresholds. Applying the age-adjusted thresholds resulted in a considerable reduction (6.0–36.3%) of subjects fulfilling the blood pressure criteria. Especially fewer young men met the criteria for this feature, meaning that there is a large group of men with a blood pressure range of 130–140 systolic and 85–90 diastolic. Whether this group of men face severe long-term implications needs further investigation.

Several long-term follow-up studies have shown that cardiovascular risk gradually increases with rising blood pressure (41, 42, 43, 44). Treatment with blood pressure-lowering medications has been shown to be beneficial in reducing the incidence of cardiovascular events (45). In our study, we observed that the prevalence of subjects meeting the age-adjusted blood pressure thresholds better approached the prevalence estimates of subjects treated for hypertension. However, still some under-treatment was observed within all BMI and age groups, especially among young men. Since we are limited to cross-sectional data of Lifelines, we could not establish a cause for this. However, it may be that fewer men are checked for elevated blood pressure. Indeed, it has been reported that men are less likely than women to receive certain preventive services (46, 47, 48).

The ‘elderly’ is a difficult definition, because this subgroup is not a simple age range, but includes groups with a different level of overall health. Treatment of hypertension is therefore more complex in the elderly compared to the general population. Lowering systolic blood pressure below 140 mmHg or even below 130 mmHg to reduce cardiovascular risk is supported by data from respectively, the HOT study and the INVEST study (49, 50). Although the elderly people may benefit from antihypertensive treatment as well, it was shown previously that among ten placebo-controlled trials only one trial achieved an average systolic blood pressure value <140 mmHg in the elderly (51). In a study among 1.25 million people, with a median follow-up time of 5.2 years, the relative risks for nearly all CVD decreased with age when systolic and diastolic blood pressure increased with respectively, 20/10 mmHg (52). This indicates that a very strict blood pressure target seems less useful in older subjects compared to applying a strict blood pressure target in younger subjects. While this finding was supported by the SHEP study (53), two Japanese trials in older patients were underpowered to observe benefits from more- (<140 mmHg) vs less- (<150 mmHg) intensive blood pressure lowering on composites of cardiovascular events (54, 55).

While at first sight the decision of the JNC 8 to recommend age-specific treatment targets is in line with the available evidence, there is some criticism as well. The JNC 8 mainly used data from randomized clinical trials, while evidence from observational studies, systematic reviews or meta-analyses were excluded (40). Still, well-conducted trials are needed to investigate the size of benefits of treating the elderly with mild hypertension (140–159 systolic and 90–99 diastolic).

### Strengths and limitations

There are several strong points, which characterize our study. We used data of 74,531 Dutch participants, of only Western European descent, from whom high-quality data on anthropometric and clinical measurements were obtained. The large number of participants allowed us to explore trends within detailed clusters of sex, BMI and age, which has not been done before. However, the findings of our study are limited by the cross-sectional data, and therefore, no trends in the development of clinically significant endpoints, such as T2D and cardiovascular morbidity and mortality, could yet be established. Although Lifelines is a relatively young cohort, it is one of the largest cohort studies to date, which is prospectively collecting follow-up data on a wide range of subjects. The Lifelines Cohort Study will therefore add a valuable contribution to strengthen evidence upon complex research questions.

## Conclusion

In this representative sample of a Dutch adult population, we observed a gender disparity with age and BMI for the prevalence of MetS and, especially, the blood pressure and waist circumference component. The observed sex differences tended to diminish in older adults. Due to the strict selected threshold level in the R-ATPIII, the blood pressure component is much higher in the (elderly) population compared to the other MetS components. This update of the MetS prevalence and its individual components in the Dutch population show that there is an ongoing burden of risk factors associated with development of T2D and CVD.

## Supplementary data

Table S1 Clinical characteristics of the normal weight population.

## Declaration of interest

The authors declare that there is no conflict of interest that could be perceived as prejudicing the impartiality of the research reported.

## Funding

The Lifelines Cohort Study, and generation and management of GWAS genotype data for the Lifelines Cohort Study is supported by the Netherlands Organization of Scientific Research NWO (grant 175.010.2007.006), the Ministry of Economic Affairs, the Ministry of Education, Culture and Science, the Ministry for Health, Welfare and Sports, the Northern Netherlands Collaboration of Provinces (S N N), the Province of Groningen, University Medical Center Groningen, the University of Groningen, Dutch Kidney Foundation and Dutch Diabetes Research Foundation. This work was supported by the National Consortium for Healthy Ageing, and funds from the European Union’s Seventh Framework program (FP7/2007–2013) through the BioSHaRE-EU (Biobank Standardisation and Harmonisation for Research Excellence in the European Union) project, grant agreement 261433. J V van Vliet-Ostaptchouk was supported by a Diabetes Funds Junior Fellowship from the Dutch Diabetes Research Foundation (project no. 2013.81.1673). Lifelines (BRIF4568) is engaged in a Bioresource research impact factor (BRIF) policy pilot study, details of which can be found at: https://www.bioshare.eu/content/bioresource-impact-factor.
