# Supplementary material for: Sex, BMI and age differences in metabolic syndrome: the Dutch Lifelines Cohort Study
Source: Endocr Connect. 2017 Apr 18;6(4):278–88. doi: 10.1530/EC-17-0011 (PMC5457493; doi:10.1530/EC-17-0011)
Supplement: Table S1 [file ec-6-278-t001.pdf]

Sex, BMI and age differences in metabolic syndrome:
The Dutch Lifelines Cohort Study.

Supplemental information

Supplemental table 1A. Clinical characteristics of the normal weight population.

**Men**	**18-29**	**30-39**	**40-49**	**50-59**	**60-69**	**70-79**
Number of subjects	*2,507*	*3,244*	*4,002*	*1,600*	*976*	*362*
Systolic BP, mmHg	124 ± 11	125 ± 11	126 ± 12	127 ± 14	132 ± 16	137 ± 19
Diastolic BP, mmHg	68 ± 7	72 ± 7	76 ± 8	77 ± 9	77 ± 9	76 ± 9
Waist circumference, cm	82.2 ± 6.0	85.6 ± 5.9	87.4 ± 5.9	88.5 ± 6.0	89.1 ± 6.0	90.8 ± 5.8
HDL-C, mmol/L	1.37 ± 0.28	1.37 ± 0.31	1.42 ± 0.33	1.47 ± 0.34	1.51 ± 0.35	1.48 ± 0.35
Triglycerides, mmol/L	0.85 (0.65-1.14)	0.94 (0.69-1.31)	0.98 (0.73-1.37)	1.01 (0.75-1.39)	0.98 (0.75-1.29)	0.94 (0.73-1.23)
Fasting blood glucose, mmol/L	4.8 ± 0.6	4.9 ± 0.6	5.0 ± 0.7	5.1 ± 0.8	5.1 ± 0.8	5.3 ± 0.8
Use of anti-hypertensive drugs (%)	0.4	1.0	2.6	4.9	17.2	35.1
Type 2 diabetes (%)	0.3	0.4	0.7	1.3	3.6	4.9
CVD history (%)	0.4	0.2	0.5	1.2	4.6	12.4
**Women**	**18-29**	**30-39**	**40-49**	**50-59**	**60-69**	**70-79**
Number of subjects	*3,474*	*5,260*	*7,833*	*3,006*	*1,453*	*434*
Systolic BP, mmHg	114 ± 11	114 ± 12	118 ± 13	122 ± 15	130 ± 18	138 ± 19
Diastolic BP, mmHg	67 ± 7	69 ± 8	71 ± 8	72 ± 9	72 ± 9	72 ± 9
Waist circumference, cm	76.2 ± 7.0	78.1 ± 6.8	79.3 ± 6.8	80.7 ± 7.0	81.4 ± 7.0	82.7 ± 7.3
HDL-C, mmol/L	1.57 ± 0.35	1.62 ± 0.35	1.73 ± 0.38	1.85 ± 0.43	1.85 ± 0.44	1.85 ± 0.45
Triglycerides, mmol/L	0.78 (0.60-1.03)	0.70 (0.55-0.93)	0.75 (0.59-0.98)	0.84 (0.65-1.09)	0.92 (0.73-1.23)	0.96 (0.74-1.25)
Fasting blood glucose, mmol/L	4.6 ± 0.4	4.6 ± 0.4	4.7 ± 0.5	4.8 ± 0.5	5.0 ± 0.7	5.1 ± 1.0
Use of anti-hypertensive drugs (%)	0.6	1.4	3.5	7.0	16.9	33.6
Type 2 diabetes (%)	<0.1	0.1	0.3	0.3	2.3	5.6
CVD history (%)	0.2	0.2	0.4	0.7	1.2	3.0

Abbreviations: blood pressure, BP; high density lipoprotein cholesterol, HDL-C; cardiovascular disease, CVD.

Supplemental table 1B. Clinical characteristics of the overweight population.

**Men**	**18-29**	**30-39**	**40-49**	**50-59**	**60-69**	**70-79**
Number of subjects	*1,154*	*3,262*	*5,974*	*2,636*	*1,893*	*758*
Systolic BP, mmHg	129 ± 11	130 ± 11	131 ± 13	133 ± 14	137 ± 16	141 ± 17
Diastolic BP, mmHg	70 ± 7	75 ± 8	79 ± 9	80 ± 9	80 ± 9	78 ± 9
Waist circumference, cm	93.2 ± 6.2	95.7 ± 5.9	97.3 ± 6.0	98.4 ± 6.0	99.3 ± 6.1	100.2 ± 6.2
HDL-C, mmol/L	1.23 ± 0.27	1.21 ± 0.26	1.25 ± 0.28	1.30 ± 0.31	1.34 ± 0.32	1.32 ± 0.31
Triglycerides, mmol/L	1.09 (0.80-1.56)	1.24 (0.89-1.81)	1.30 (0.93-1.88)	1.25 (0.94-1.77)	1.18 (0.89-1.62)	1.15 (0.87-1.57)
Fasting blood glucose, mmol/L	4.9 ± 0.7	5.1 ± 0.7	5.2 ± 0.7	5.3 ± 0.8	5.4 ± 0.9	5.6 ± 1.1
Use of anti-hypertensive drugs (%)	0.4	2.0	5.3	10.3	30.5	51.3
Type 2 diabetes (%)	0.3	0.9	1.4	1.5	7.9	11.8
CVD history (%)	<0.1	0.3	1.0	2.0	7.3	14.8
**Women**	**18-29**	**30-39**	**40-49**	**50-59**	**60-69**	**70-79**
Number of subjects	*1,142*	*2,684*	*5,014*	*2,519*	*1,808*	*726*
Systolic BP, mmHg	119 ± 11	119 ± 11	123 ± 13	127 ± 16	133 ± 17	140 ± 18
Diastolic BP, mmHg	69 ± 7	71 ± 8	74 ± 9	74 ± 9	74 ± 9	74 ± 9
Waist circumference, cm	88.0 ± 7.4	89.1 ± 7.1	89.9 ± 7.0	90.9 ± 7.0	92.0 ± 6.9	93.2 ± 6.9
HDL-C, mmol/L	1.42 ± 0.33	1.47 ± 0.32	1.54 ± 0.35	1.64 ± 0.38	1.64 ± 0.38	1.63 ± 0.39
Triglycerides, mmol/L	0.87 (0.67-1.16)	0.82 (0.62-1.12)	0.90 (0.69-1.24)	1.00 (0.76-1.38)	1.12 (0.85-1.53)	1.19 (0.91-1.58)
Fasting blood glucose, mmol/L	4.7 ± 0.5	4.8 ± 0.7	4.9 ± 0.7	5.0 ± 0.6	5.2 ±0.9	5.4 ±1.0
Use of anti-hypertensive drugs (%)	1.0	2.5	6.4	9.9	28.4	50.6
Type 2 diabetes (%)	0.3	0.2	0.2	0.8	5.1	9.5
CVD history (%)	0.2	0.3	0.3	0.5	3.0	4.4

Abbreviations: blood pressure, BP; high density lipoprotein cholesterol, HDL-C; cardiovascular disease, CVD.

Supplemental table 1C. Clinical characteristics of the obese population.

**Men**	**18-29**	**30-39**	**40-49**	**50-59**	**60-69**	**70-79**
Number of subjects	*241*	*857*	*1,734*	*780*	*578*	*173*
Systolic BP, mmHg	135 ± 13	135 ± 12	137 ± 14	137 ± 14	141 ± 17	141 ± 19
Diastolic BP, mmHg	73 ± 8	78 ± 8	81 ± 9	82 ± 9	81 ± 9	76 ± 8
Waist circumference, cm	109.7 ± 9.0	110.0 ± 9.0	111.6 ± 8.8	111.5 ± 8.6	113.0 ± 8.1	112.4 ± 7.6
HDL-C, mmol/L	1.07 ± 0.24	1.09 ± 0.24	1.12 ± 0.26	1.14 ± 0.25	1.21 ± 0.27	1.21 ± 0.31
Triglycerides, mmol/L	1.43 (1.02-1.90)	1.62 (1.15-2.26)	1.60 (1.16-2.30)	1.60 (1.17-2.25)	1.44 (1.09-1.98)	1.36 (1.04-1.78)
Fasting blood glucose, mmol/L	5.1 ± 4.9	5.2 ± 0.7	5.5 ± 1.1	5.7 ± 1.1	5.9 ± 1.3	6.1 ± 1.4
Use of anti-hypertensive drugs (%)	1.7	4.6	12.3	21.8	45.0	68.2
Type 2 diabetes (%)	0.8	1.6	6.2	9.7	18.5	28.9
CVD history (%)	0.0	1.2	1.7	4.0	9.2	22.0
**Women**	**18-29**	**30-39**	**40-49**	**50-59**	**60-69**	**70-79**
Number of subjects	*493*	*1,323*	*2,427*	*1,009*	*823*	*372*
Systolic BP, mmHg	123 ± 11	123 ± 12	129 ± 15	132 ± 15	136 ± 17	139 ± 17
Diastolic BP, mmHg	70 ± 7	73 ± 8	76 ± 9	76 ± 9	75 ± 9	73 ± 8
Waist circumference, cm	102.2 ± 10.5	103.6 ± 10.5	105.0 ± 10.5	104.9 ± 10.1	105.2 ± 9.3	105.4 ± 9.4
HDL-C, mmol/L	1.29 ± 0.30	1.32 ± 0.31	1.38 ± 0.32	1.45 ± 0.35	1.50 ± 0.35	1.47 ± 0.35
Triglycerides, mmol/L	1.03 (0.78-1.33)	1.00 (0.76-1.37)	1.14 (0.84-1.57)	1.27 (0.95-1.81)	1.31 (0.97-1.75)	1.39 (1.10-1.76)
Fasting blood glucose, mmol/L	4.9 ± 0.7	5.0 ± 0.8	5.2 ± 0.9	5.5 ± 1.1	5.7 ± 1.2	5.9 ± 1.6
Use of anti-hypertensive drugs (%)	2.4	4.2	13.5	26.5	48.0	68.5
Type 2 diabetes (%)	0.6	1.4	3.9	7.8	15.7	20.7
CVD history (%)	0.0	0.4	0.7	1.5	3.3	7.5

Abbreviations: blood pressure, BP; high density lipoprotein cholesterol, HDL-C; cardiovascular disease, CVD.

Supplemental table 2A. The prevalence of the individual MetS components among the normal weight population.

**Men**	**18-29**	**30-39**	**40-49**	**50-59**	**60-69**	**70-79**
Number of subjects	*2,507*	*3,244*	*4,002*	*1,600*	*976*	*362*
Waist circumference	0.1	0.4	0.5	1.5	1.4	3.0
Blood pressure	29.3	33.9	38.8	43.4	60.8	76.0
HDL-cholesterol	11.3	14.8	11.6	8.8	7.2	7.2
Triglycerides	7.1	12.0	14.2	15.2	9.8	9.7
Fasting blood glucose	3.4	5.2	9.7	13.7	17.7	23.8

**Women**	**18-29**	**30-39**	**40-49**	**50-59**	**60-69**	**70-79**
Number of subjects	*3,474*	*5,260*	*7,833*	*3,006*	*1,453*	*434*
Waist circumference	6.0	9.3	11.9	17.1	19.6	24.9
Blood pressure	8.7	10.0	19.3	32.6	55.1	73.7
HDL-cholesterol	16.8	13.4	8.5	6.5	6.8	9.9
Triglycerides	3.3	2.7	3.4	6.2	8.4	10.1
Fasting blood glucose	1.1	1.8	3.5	5.1	11.5	17.7

Supplemental table 2B. The prevalence of the individual MetS components among the overweight population.

**Men**	**18-29**	**30-39**	**40-49**	**50-59**	**60-69**	**70-79**
Number of subjects	*1,154*	*3,262*	*5,974*	*2,636*	*1,893*	*758*
Waist circumference	9.5	16.6	24.2	28.0	35.5	39.6
Blood pressure	48.4	50.1	56.1	62.9	76.9	87.6
HDL-cholesterol	25.9	27.6	25.2	20.4	17.8	19.8
Triglycerides	18.8	28.6	31.1	27.9	22.1	21.2
Fasting blood glucose	4.8	11.4	17.1	23.4	31.3	37.6

**Women**	**18-29**	**30-39**	**40-49**	**50-59**	**60-69**	**70-79**
Number of subjects	*1,142*	*2,684*	*5,014*	*2,519*	*1,808*	*726*
Waist circumference	51.0	57.0	62.4	67.7	73.3	77.8
Blood pressure	17.2	18.3	32.7	43.9	66.4	85.0
HDL-cholesterol	31.8	26.1	20.4	14.3	13.2	14.0
Triglycerides	7.1	5.7	9.8	13.7	18.2	19.3
Fasting blood glucose	2.3	3.9	8.3	12.3	21.5	27.7

Supplemental table 2C. The prevalence of the individual MetS components among the obese population.

**Men**	**18-29**	**30-39**	**40-49**	**50-59**	**60-69**	**70-79**
Number of subjects	*241*	*857*	*1,734*	*780*	*578*	*173*
Waist circumference	85.5	84.9	91.2	91.0	95.5	95.4
Blood pressure	68.5	66.4	74.7	78.5	87.7	92.5
HDL-cholesterol	52.3	46.6	41.5	38.8	29.2	28.3
Triglycerides	31.5	47.3	46.5	44.4	35.5	31.8
Fasting blood glucose	10.8	19.1	33.6	40.5	52.9	54.3

**Women**	**18-29**	**30-39**	**40-49**	**50-59**	**60-69**	**70-79**
Number of subjects	*493*	*1,323*	*2,427*	*1,009*	*823*	*372*
Waist circumference	92.7	96.1	97.8	97.5	98.5	98.9
Blood pressure	29.8	30.9	51.7	65.6	83.0	93.5
HDL-cholesterol	49.9	45.9	37.6	30.7	26.4	25.0
Triglycerides	12.8	13.2	20.0	28.6	27.7	28.2
Fasting blood glucose	5.7	10.1	21.3	33.6	42.9	47.3
